# Anomalous hydrogen evolution behavior in high-pH environment induced by locally generated hydronium ions

**DOI:** 10.1038/s41467-019-12773-7

**Published:** 2019-10-25

**Authors:** Xuesi Wang, Chaochen Xu, Mietek Jaroniec, Yao Zheng, Shi-Zhang Qiao

**Affiliations:** 1 School of Chemical Engineering, The University of Adelaide, Adelaide, SA 5005 Australia; 2 Department of Chemistry and Biochemistry, Kent State University, Kent, OH 44242 USA

**Keywords:** Catalytic mechanisms, Electrochemistry, Materials for energy and catalysis, Nanoscale materials

## Abstract

Most fundamental studies of electrocatalysis are based on the experimental and simulation results obtained for bulk model materials. Some of these mechanistic understandings are inapplicable for more active nanostructured electrocatalysts. Herein, considering the simplest and most typical electrocatalytic process, the hydrogen evolution reaction, an alternative reaction mechanism is proposed for nanomaterials based on the identification of a new intermediate, which differs from those commonly known for the bulk counterparts. In-situ Raman spectroscopy and electrochemical thermal/kinetic measurements were conducted on a series of nanomaterials under different conditions. In high-pH electrolytes with negligible hydronium (H_3_O^+^) concentration in bulk phase, massive H_3_O^+^ intermediates are found generating on the catalytic surface during water dissociation and hydrogen adsorption processes. These H_3_O^+^ intermediates create a unique acid-like local reaction environment on nanostructured catalytic surfaces and cut the energy barrier of the overall reaction. Such phenomena on nanostructured electrocatalysts explain their widely observed anomalously high activity under high-pH conditions.

## Abstract (web summary)

Most of the current understanding on electrocatalysis is obtained on bulk catalysts but has not been fully verified on nanostructured materials. An alternative alkaline hydrogen evolution reaction mechanism is proposed here for nanostructured catalysts.

## Introduction

Heterogeneous electrocatalytic reduction reactions, e.g., water, nitrogen, and carbon dioxide reduction, are attracting more attention due to their importance for production of hydrogen, carbon monoxide/methane, and ammonia through simple electrochemical hydrogenation processes^1–7^. To carry out these reactions under mild and cost-effective conditions, water-based alkaline solutions are usually used as the hydrogen source. During these reactions, water is not only a solvent but is also usually dissociated into H* and OH* species (asterisk (*) represents an active site on the catalyst), which further interact with other reactive intermediates through proton-coupled electron transfer to generate the final products^2,4,8–12^. As a result, it is important to reveal the local environments about the reactant, reaction intermediates, and catalyst surfaces. So far, the understanding of this complicated issue in electrocatalysis is mostly achieved based on the experimental and simulation studies for uniform single-/poly-crystal models^13–22^. These fundamental studies were always carried out for alkaline hydrogen evolution reaction (HER) as a simple model that involves both water dissociation and proton reduction processes. Most results showed that alkaline HER activity of a single-/poly-crystal material is dependent on the pH value of the electrolyte, i.e., the HER kinetics gradually decreases with increasing pH^15,18,23^. This may be due to pH-dependent H adsorption energy and/or water dissociation energy barrier^15,24–26^. More importantly, such mechanism suggests a sluggish HER kinetics on bulk materials in high-pH environments (e.g., 1 M KOH), largely limiting their practical applications in the aforementioned reduction reactions.

On the other hand, owing to the high specific surface area and a variety of active sites, nanomaterials are more efficient and cost-effective as heterogeneous electrocatalysts. Thus the design of more active nanostructured electrocatalysts is now the key research concept^27^. However, when the reaction mechanism is taken into account, the well-known principles established for the bulk materials are sometimes inapplicable for nanostructured electrocatalysts^20^. One of the well-known facts is that the HER activity of many nanostructured electrocatalysts is much better in high-pH (0.1–1 M KOH, pH = 13–13.5) electrolytes than in less-alkalic environments (0.01 M KOH to neutral buffer, pH = 12–7.1)^28–33^. This is opposite to the HER activity trend observed for single-/poly-crystal metals (e.g., activity decreases with raising pH). This different behavior of nanostructured and bulk electrocatalysts indicates not only different HER mechanism on these catalysts but, more importantly, also suggests that the water dissociation and proton adsorption/reduction on the surface of nanostructured electrocatalysts are more complicated than those on bulk metals. However, very little research has been conducted toward distinguishing the difference in the reaction mechanisms on the bulk and nanostructured electrocatalysts. As a result, the design principle of nanostructured electrocatalysts for reduction reactions in aqueous electrolytes is still mainly based on the “inappropriate” knowledge of the bulk materials.

Herein we report a new insight into HER process on the surface of nanostructured electrocatalysts different from that for the well-understood bulk materials, specifically at high-pH electrolytes that contain a negligible amount of hydronium ions (H_3_O^+^) in the bulk aqueous solutions. This study shows that H_3_O^+^ ions are in situ generated on the nanostructured Pt-based catalyst’s surface during the HER process, inducing an acidic local surface environment. As a result, these catalysts exhibit an anomalous acid-like HER activity at high-pH electrolytes, e.g., high activity with acid-like Tafel slope of ~30 mV dec^−1^ and low activation energy. Formation of the favorable H_3_O^+^ layer is most likely due to the high-rate water dissociation process that results in a large amount of free H_3_O^+^ within the electric double layer of nanostructured electrodes, confirmed by in situ Raman spectroscopy and electrochemical thermal and kinetic analysis. This alternative mechanism as compared to that on the bulk materials sheds a new light toward the design of electrocatalytic nanomaterials by taking the effect of local intermediates in aqueous electrolytes into account.

## Results

### HER kinetics

**Fig. 1:**
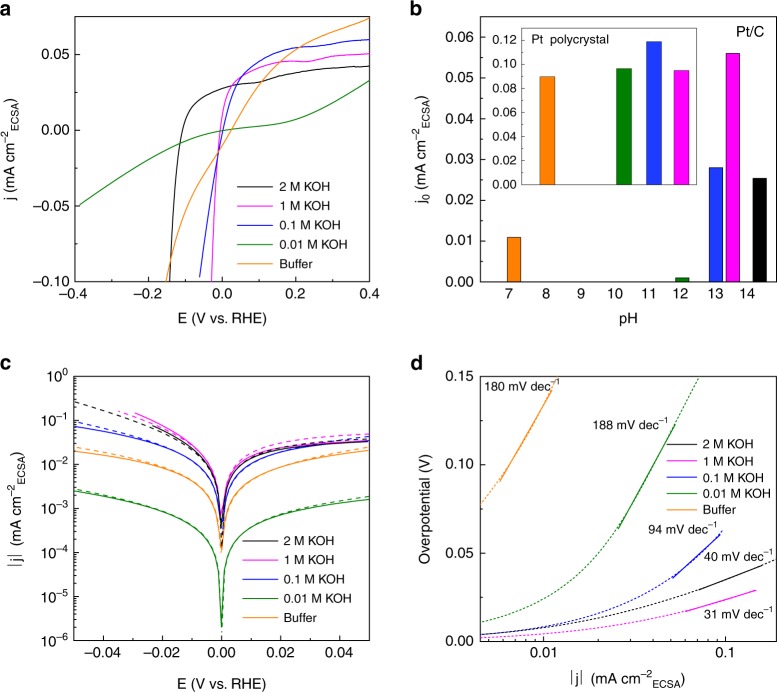
HER activity comparison under different alkaline environments. **a** The HER polarization curves of Pt/C under different conditions. **b** The activity trend for Pt/C and polycrystal Pt (inset) under different conditions. The data for polycrystal Pt were taken from ref. ^35^. **c** Tafel plots and the corresponding Butler–Volmer fitting results for Pt/C under different conditions. **d** The Tafel slope for Pt/C under different conditions

In correspondence to the mechanical studies carried out on bulk Pt and its alloys, we use the standard Pt/C and a series of Pt-based bimetallic nanomaterials as models to reveal the nature of water dissociation and proton reduction on nanostructured electrocatalysts^34^. Characterization of these materials (including transmission electron microscopic images and X-ray powder diffraction patterns) provided in Supporting Information (Supplementary Figs. 1 and 2) shows that the differences in their structural properties are tiny. Figure 1a shows the HER polarization curves recorded for Pt/C (20 wt % metal) in electrolytes from neutral buffer (pH = 7.1) to 1 M KOH (pH = 13.5). It can be seen that the apparent activity of Pt/C is significantly higher under high OH^−^ concentration ([OH^−^]) in comparison to that under less alkaline environment. Note that the low activity in 2 M solution may be due to the slower kinetics of OH^*^ transfer in such highly alkaline environment than the rate of water dissociation. In addition, Pt/C’s activity in 1 M KOH is much higher than those in other conditions, the trend of which is very different from the previous results obtained for bulk Pt electrode (Fig. 1b)^15,25,35^. Further, the rate-determining step (RDS) of HER is also various in different alkaline electrolytes, which is evidenced in the Tafel plots shown in Fig. 1c, d. Tafel slopes in different electrolytes are compared in similar range (~50 mV negative than the equilibrium potential) to avoid the influence from the high overpotential polarization and formed bubbles. The Tafel slope for Pt/C is ~180 mV dec^−1^ in buffer and 0.01 M KOH solutions, indicating that in these environments RDS for HER is water dissociation process. However, the Tafel slope drops to 94 mV dec^−1^ when [OH^−^] increases to 0.1 M, while in 1 M KOH solution, the Tafel slope for HER on Pt/C is around 30 mV dec^−1^ (~40 mV dec^−1^ in 2 M KOH solution). For these reactions with a Tafel slope under 120 mV dec^−1^, the RDS of the reaction is either proton recombination (Volmer–Tafel) step or electrochemical desorption (Volmer–Heyrovsky) step, similar to the RDS of Pt/C in acid environments. Both reaction pathways indicate that there is a sufficient amount of hydronium ions on the electrode surface for HER process. Otherwise, water dissociation should be the RDS of the reaction, yielding a Tafel slope >120 mV dec^−1^. Such phenomena are also observed in LiOH-based electrolytes (Supplementary Fig. 3), indicating that the small Tafel slope in high-pH environment is not induced by a specific cation but generally exists in different types of alkaline solutions. In addition, these results can be observed on other Pt-based nanomaterials as well (Supplementary Figs. 4–6), further confirming that the nanostructured electrocatalysts have an acid-like RDS for HER in high-pH environments.

### HER activity trends

**Fig. 2:**
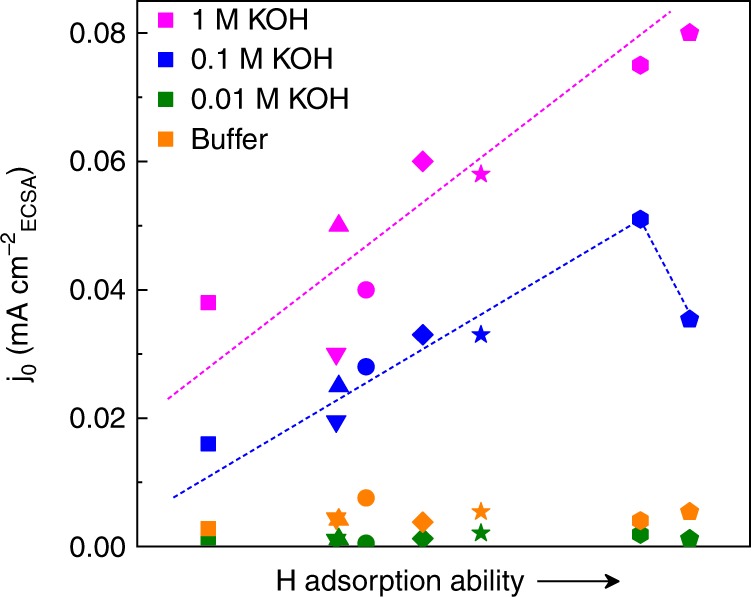
The experimentally acquired relationship between the H adsorption ability and the activity (*j*_0_) for a series of Pt-based materials using different electrolytes. The symbols in the figure are circle: Pt/C; square: PtFe/C; standing triangle: PtCo@Pt/C; inverted triangle: PtCo/C; diamond: PtNi/C; star: PtFe@Pt/C; pentagon: dealloyed PtNi/C; hexagon: PtNi@Pt/C. The H adsorption ability of the electrocatalysts is represented according to the d-band vacancies of each material

As the HER activity is closely dependent on the H adsorption ability of the materials, we investigated the change of H adsorption for a series of Pt-based nanomaterials under different [OH^−^] conditions. Here the experimentally obtained d-band vacancies (calculated through the ex situ X-ray absorption near-edge structure spectra (XANES) shown in Supplementary Fig. 7) are used to represent the H adsorption ability of the catalysts; the larger vacancy of the Pt’s d-band, the stronger Pt-H bond, and vice versa^36,37^. Afterwards, by quantitatively evaluating the H adsorption ability of a group of different Pt-based nanomaterials, we obtained a relationship between the H adsorption ability and the material’s activity^34^. As can be seen in Fig. 2a, the activities of all these catalysts in different electrolytes follow the order: 1 M KOH > 0.1 M KOH » neutral buffer > 0.01 M KOH. This confirms that the nanostructured electrocatalysts studied exhibit anomalously high HER activities in high-pH solutions. More importantly, it can be seen that a volcano-type relationship exists between the H adsorption ability and the activity of the materials in 1 M (only the left side of the volcano plot was achieved for the catalysts tested in 1 M solution) and 0.1 M KOH electrolytes. Such volcano relationship is not observed in 0.01 M KOH and neutral buffer electrolytes (Supplementary Fig. 8). It is well known that, in an acid environment, the HER activity of a catalyst is solely depending on its hydrogen adsorption ability that has a very close relationship with catalyst’s electronic structure (e.g., d-band vacancy)^38–40^. However, in an alkaline environment, the HER activity of a catalyst is more dependent on other factors (e.g., water dissociation rate) and not only on the hydrogen adsorption ability. Normally, for the bulk metal surfaces the relationship between hydrogen adsorption ability and catalysts’ HER activities does not match the volcano shape in alkaline environments well^41^. Thus the observed volcano relationship between the H adsorption ability of the catalysts and their activities in high-pH electrolyte indicates that the HER behavior of the nanostructured electrocatalysts under such conditions is more like that in the acid environment.

### In situ Raman characterization

**Fig. 3:**
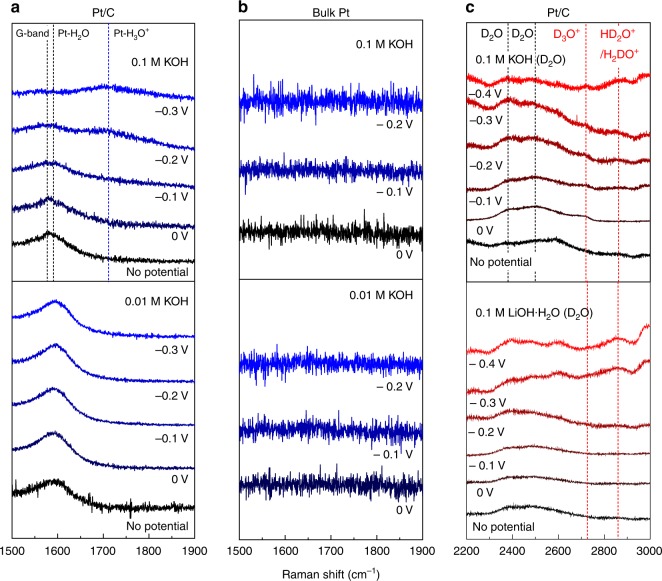
Raman spectra of Pt/C and bulk Pt at various conditions. The Raman spectra for: **a** Pt/C and **b** bulk Pt in water-based alkaline environments. **c** Pt/C in deuterium water-based alkaline environments. The marked overpotential is in comparison to the onset potential of HER (e.g., −0.1 V is 0.1 V more negative to onset potential). The Raman signals on the surface of the catalyst are identified as: G-band of carbon: ~1580 cm^−1^; H_2_O: ~1600 cm^−1^; H_3_O^+^: ~1750 cm^−1^; D_3_O^+^: ~2720 cm^−1^; HD_2_O/H_2_DO^+^: ~2850 cm^−1^; D_2_O: ~2380 cm^−1^, ~2500 cm^−1^^43–45^

We used in situ Raman spectra to detect the adsorbates on the catalysts in different alkaline environments and support our above assumption^42^. The samples used in Raman testing were freshly prepared and Nafion was not used in the ink (5 mg mL^−1^ catalyst–water solution without any binder) to avoid the presence of an additional acid-spectator. To rule out the influence from the bulk solution, the reference spectra (with no potential applied) were taken for each group of samples, on which no peaks of electrochemically adsorbed intermediates can be observed. As shown in Fig. 3a and Supplementary Fig. 9, a series of peaks such as Pt-H (~2100 cm^−1^)^43^, H_2_O (~1600 cm^−1^), and H_3_O^+^ (~1750 cm^−1^) is observed on the spectra of nanostructured Pt/C surfaces at high-pH electrolyte. The positions of these peaks are consistent with the theoretical values and/or reported data^43–45^. However, such features are absent on the spectra of bulk Pt samples (Fig. 3b) and Pt/C in 0.01 M solution (Fig. 3a). Specifically, as can be seen from Supplementary Fig. 9, the H intermediates are already adsorbed on the Pt/C catalyst before the onset potential (0 V) of HER and soon get desorbed when a negative potential is applied. Meanwhile, the original G-band peak of the materials (~1590 cm^−1^) also becomes broader with increasing potential as compared to the reference curve due to the large amount of adsorbed water (Fig. 3a)^44^. Interestingly, the Pt–H_3_O^+^ interaction peak appears and becomes stronger with increasing overpotential (Fig. 3a). Note that the concentration of H_3_O^+^ species in the bulk of all tested alkaline electrolytes is extremely low (10^−13^ M). Thus the H_3_O^+^ species detected by Raman spectra on the surface of catalysts have to be in situ generated during the HER process. So far, the interaction between Pt and H_3_O^+^ was only reported on the bulk Pt in acid environments and was not observed on the bulk Pt in any alkaline solutions^44,46^. With such strong H_3_O^+^ signal detected here, one can assume that an acid-like environment with rich H_3_O^+^ species is created on the catalyst surface in a high-pH environment. Such formation of H_3_O^+^ during HER process in 0.1 M KOH solution is not only observed on Pt/C but also on other nanomaterials. As highly active nanostructured HER electrocatalysts, PtNi/C and dealloyed PtCo/C were also examined in different alkaline environments. For both materials, Pt-H_3_O^+^ features can be detected at around −0.1 V in 0.1 M KOH electrolytes (Supplementary Fig. 10). Moreover, the existence of H_3_O^+^ was further confirmed by deuterium substitution experiment with both LiOH and KOH electrolytes. As can be seen in Fig. 3c, the corresponding D_3_O^+^ (~2720 cm^−1^) and HD_2_O^+^/H_2_DO^+^(~2850 cm^−1^) signals can be observed on the spectra of nanostructured Pt/C in 0.1 M solution^45^. In comparison, such signals are not observed on the spectra of bulk Pt and any other nanostructured electrocatalysts when the reaction takes place in 0.01 M KOH solution in either H_2_O or D_2_O environment (Supplementary Figs. 11–13). This indicates that the Pt–H_3_O^+^ interaction is distinctive for high-pH environments.

### Interaction between electrocatalyst and intermediates

**Fig. 4:**
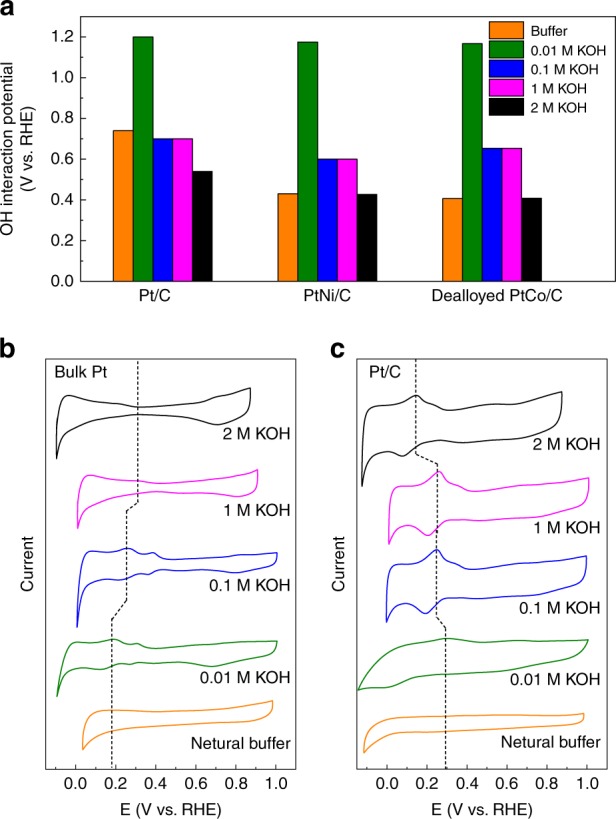
The trends of H and OH interactions with different catalysts. **a** OH interaction potentials obtained for three catalysts in different electrolytes. Data obtained from CO stripping measurements. **b**, **c** CVs of bulk Pt and Pt/C in different electrolytes. The dotted line indicates the shifting trend of the H_upd_ peak

We propose that the formation of H_3_O^+^ is closely related to the generation of H* and water dissociation process in the reaction. It is well known that water dissociation can be facilitated by good interaction between OH intermediates and the catalytic surface. In addition, such interaction can be monitored using carbon monoxide (CO) stripping tests^47–50^. Qualitatively, a lower CO oxidation potential suggests stronger interaction between the catalyst and the OH^*^, indicating better water dissociation proceeds. As shown in Fig. 4a, Pt/C and other nanostructured electrocatalysts show much stronger interactions (lower oxidation potential) with OH^−^ with increasing [OH^−^], indicating that the water dissociation process is improved in high-pH electrolytes on these nanomaterials.

Afterwards, the interaction between the catalyst and H^*^ was studied. The previous studies conducted for bulk polycrystal Pt show that desorption of H is more difficult with growing pH in alkaline environment^17,18,25^. Such strongly adsorbed H is believed to be unbeneficial toward water reduction^17,18,25^. To compare these results with the current case, we recorded the cyclic voltammograms (CVs) for Pt/C and bulk Pt under different [OH^−^] environments. For bulk materials, the underpotentially detected H (H_upd_) peak can only be clearly observed in 0.01 M and 0.1 M KOH, with the H_upd_ potential becoming more positive with increasing [OH^−^] (Fig. 4b). When [OH^−^] is higher than 0.1 M, only a weak H_upd_ peak can be detected. This may be caused by a large amount of OH^−^ blocking some of the H adsorption sites on the bulk Pt surface. In general, the peak associated with H adsorption on the bulk Pt appears at higher potentials and shifts with increasing pH. Conversely, for Pt/C, the H_upd_ peak is shifting to the lower potentials with increasing pH, indicating that the H adsorption weakens (Fig. 4c). At this stage, it is clear that Pt/C shows the improved water dissociation ability and weakened H adsorption ability with increasing [OH^−^]. We suggest that such changes can lead to the generation of the large amount of H_3_O^+^ during the HER process; the detailed formation mechanism will be explained later.

### Activation energy

**Fig. 5:**
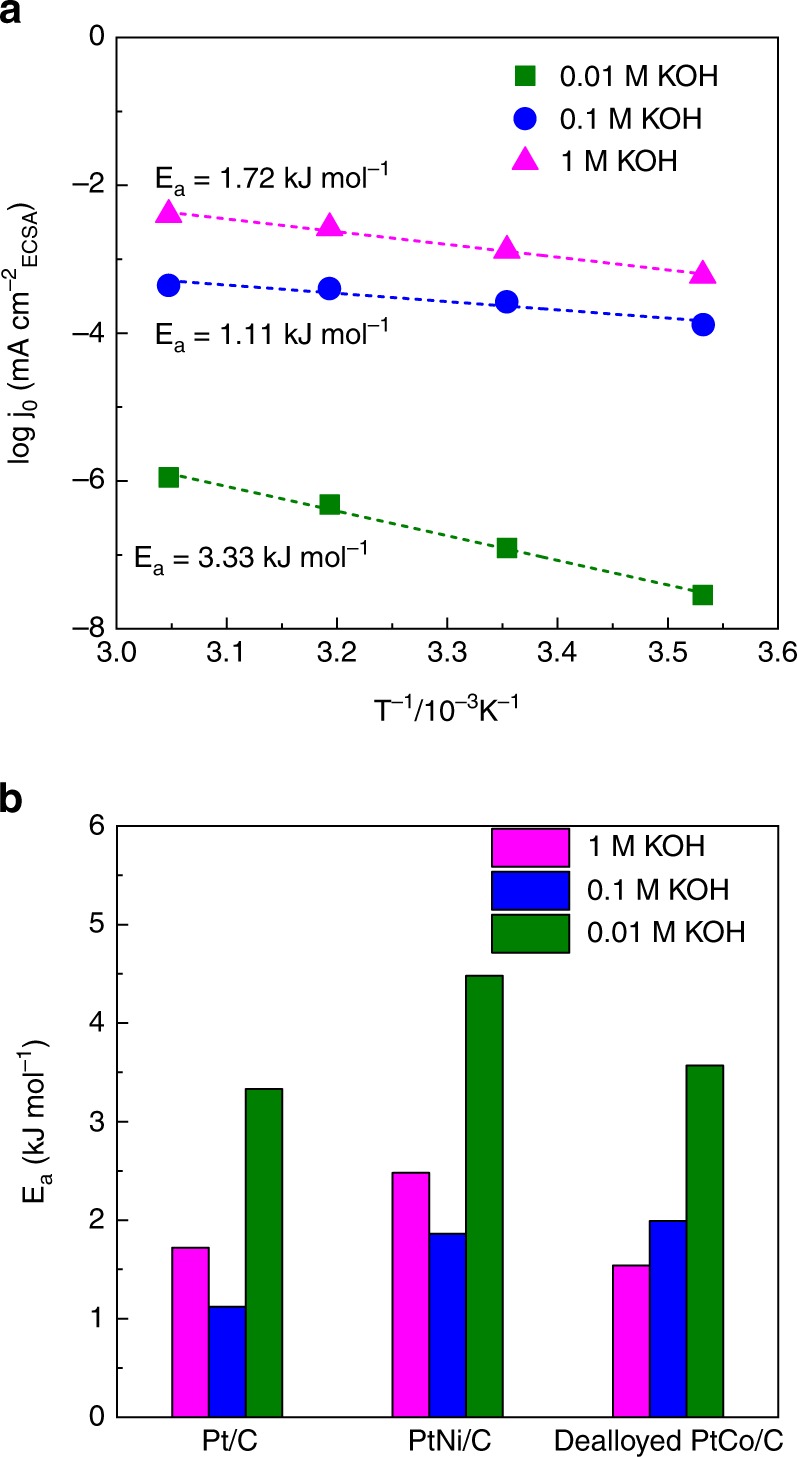
HER energy barrier for various nanostructured electrocatalysts. **a** The relationship between the temperature and the catalytic activity of Pt/C under certain temperature range (10–55 °C). **b** A comparison of the activation energy (*E*_a_) for a series of different Pt-based nanostructured electrocatalysts in different alkaline environments

The activation energy (*E*_a_) values for Pt/C and a series of Pt-based nanostructured electrocatalysts were calculated to reveal how the in situ generated H_3_O^+^ changes the overall kinetics of the reaction in different alkaline environments. The values of *E*_a_ were calculated according to the Arrhenius equation from the HER polarization curves under different temperatures (Fig. 5a and Supplementary Figs. 14 and 15)^51^. Figure 5b shows the *E*_a_ values for Pt/C, PtNi/C, and dealloyed PtCo/C under three different alkaline environments. It is clear that the values of *E*_a_ are obviously smaller at 1 M/0.1 M KOH as compared to those at 0.01 M KOH. This demonstrates that the in situ generated H_3_O^+^ intermediates lowered the energy barrier for the overall reaction, most likely by providing an acid-like environment that significantly optimizes the proton reduction process and changes RDS of the reaction.

### Origin of H_3_O^+^

**Fig. 6:**
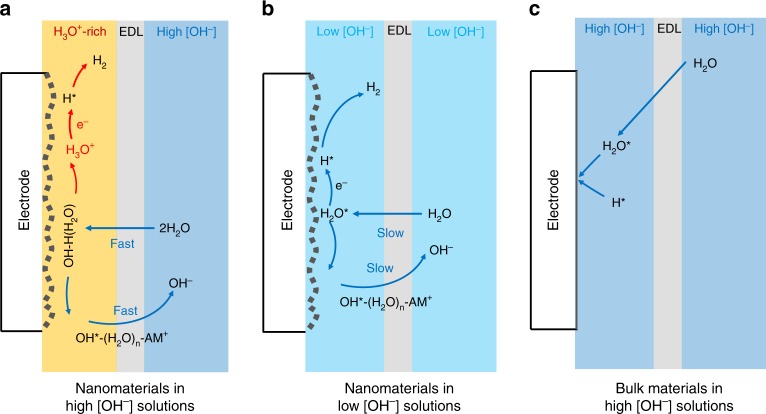
Schematic illustration of the water reduction mechanism on the nanostructured electrocatalysts. **a** Surface intermediates on the nanostructured electrocatalysts in solutions with high [OH^−^]. **b** Surface intermediates on the nanostructured electrocatalysts in solutions with low [OH^−^]. **c** Water reduction mechanism on the bulk electrocatalysts in high-pH environments. EDL electric double layer

Based on the in situ Raman spectra and electrochemical thermal/kinetic analysis, an alternative water reduction mechanism is revealed for the nanostructured Pt-based electrocatalysts. In all alkaline electrolytes, water dissociation is always the most important step toward producing hydrogen source needed for HER process. Under high [OH^−^], large amounts of OH^−^ are available in the electrolyte and can be easily adsorbed to the catalyst, strongly promoting the water dissociation process. As a result, large amounts of H^*^ are produced and the majority of the H active sites are soon covered with strongly bonded H_upd_. Note that, under negative potential, water molecules are connected to each other^52^. As water dissociation continues, more and more H ions are still bonded to nearby water molecules but not to the catalyst’s surface, which is being occupied by H_upd_ species. Thus a large amount of free H_3_O^+^ ions is generated within the double layer, resulting in an acid-like local environment. With more electrons being transferred on the surface, H_upd_ turns to H_opd_ (overpotential deposited hydrogen) and later combines with each other to form H_2_ gas (following the Tafel mechanism evidenced by a Tafel slope of ~30 mV dec^−1^), leaving an empty site. At the same time, H_3_O^+^ is reduced to H* on that site to form the cycle (Fig. 6a).

On the other hand, another dissociation product, OH intermediates, are not dissociated as OH^−^ within the double layer. According to the report by Jia et al.^53^, OH intermediates will directly form a hydroxyl–water–alkali metal cation adduct (in our case, OH^*^–(H_2_O)_*n*_–K^+^), which can be directly desorbed through the double layer into the bulk solution. As a result, the local concentration of OH^−^ does not increase and the generated OH^*^–(H_2_O)_*n*_–K^+^ does not react with hydronium species. In the current study, this assumption has also been confirmed by in situ Raman spectra. As shown in Supplementary Fig. 16, under different overpotentials, the OH stretching mode (Raman shift 3200–3600 cm^−1^) does not change much, indicating a stable status of [OH^−^] on the catalytic surface during the reaction **(**Fig. 6a).

However, the same process cannot take place in less-alkalic solutions due to the low [OH^−^] in the environment to facilitate the key water dissociation process and to supply H intermediates. In this case, Volmer step (H_2_O + e^−^ → H^*^ + OH^−^) becomes the RDS of the overall reaction as indicated by Tafel slope of 166 mV dec^−1^. High overpotential is needed for the catalysts to interact with water to start water dissociation under such low [OH^−^] environment, resulting in a high activation energy for the overall reaction. Moreover, the low concentration of metal cation also causes a slow removal of OH intermediates from the double layer. As a result, the generation of H* is sluggish, which results in the low overall HER activity (Fig. 6b).

Noticeably, this H_3_O^+^-induced water reduction mechanism seems to be unique to nanostructured electrocatalysts. This is most likely due to the complex surface structure of nanomaterials allowing the existence of a variety of different active sites to facilitate water adsorption/dissociation without interfering with the H−catalyst interactions. Since these two key processes can proceed at the same time, a facile generation of H_3_O^+^ is guaranteed. However, for uniform bulk materials with a single kind of active sites, the competition for active sites between the water dissociation and hydrogen adsorption reduces the water dissociation efficiency significantly and consequently affects the generation of hydrogen species (Fig. 6c)^24,50^. As a result, the water reduction on nanostructured electrocatalysts can be promoted by increasing [OH^−^], while hydrogen production is reduced on the bulk catalysts.

## Discussion

In summary, we studied a series of Pt-based nanostructured electrocatalysts to reveal the unique water dissociation and proton reduction mechanism on nanomaterials. A unique H_3_O^+^ intermediate layer that creates an acidic environment on the catalyst’s surface was first identified under high [OH^−^] conditions. This H_3_O^+^ intermediate layer was found to be responsible for an anomalous acid-like HER activity of nanostructured electrocatalysts in alkaline electrolytes. More electrochemical analysis and in situ Raman characterizations have indicated that these H_3_O^+^ are generated by high-rate water dissociation process that promotes desorption of H* on the surface of electrocatalysts. This unique reaction mechanism on nanomaterials may provide an important guidance for the design/selection of catalysts/electrolytes for the nanomaterial-catalyzed reactions in an aqueous environment, including carbon dioxide reduction, nitrogen reduction, and other electrocatalytic reduction reactions.

## Methods

### Fabrication of electrocatalysts

Commercialized Pt/C (20 wt.%) and PtM/C (20 wt.%, M = Fe, Co, Ni) were purchased from FuelCellStore without further treatment. The acid-treated PtM/C samples were fabricated by mixing 10 mg of PtM/C with 30 mL of HClO_4_ solution (0.1 M) and stirring overnight. The products were then washed several times and freeze-dried. The annealed PtM/C samples were fabricated by annealing 10 mg of PtM/C at 900 °C for 5 h in H_2_/Ar (H_2_ = 5 vol.%) atmosphere. All the catalysts are metal nanoparticles (size: ~5 nm) supported on carbon black.

### In situ Raman characterization

The in situ Raman spectra were recorded by HORIBA Scientific Raman Spectroscopy (laser wavelength = 532 nm) using a screen-printed chip electrode from Pine Research Instrumentation. The electrolytes were prepared with extra care to avoid contaminations from other ions and glassware. The tests were carried out using a screen-printed chip electrode from Pine Research Instrumentation. Ten microliters of the ink gel were added to the printed electrode before dried at room temperature. For the test with bulk materials, a Pt-printed chip electrode with bulk Pt surface from Pine Research Instrumentation was used.

### Electrochemical testing set-up

All the electrochemical data were recorded by a CHI 760E bipotentiostat (CH Instruments, Inc.). The powdered electrocatalysts were first dispersed in 0.05 wt.% Nafion aqueous solution to form a 2.0 mg/mL homogeneous ink gel. The working electrode was prepared by adding 20 µL of the ink gel onto the glassy carbon rotating disk electrode (surface area of the glassy carbon = 0.196 cm^2^, Pine Research Instrumentation) and dried at room temperature. The reference electrode was an Ag/AgCl wire in 4 M KCl solution. The counter electrode was a pure gold wire. All the potentials in this work were referenced to the reversible hydrogen electrode using pure hydrogen calibration and all polarization curves were current-resistance (iR) corrected. All the polarization data were represented after calibration with respect to the electrochemical active surface area. During the experiments, a flow of argon was maintained over all the CV tests, while a flow of H_2_ was purged to ensure an H_2_-oversaturated electrolyte environment during recording of all the polarization curves. The electrolytes used were KOH solution with different concentrations (0.01, 0.1, and 1 M) and phosphate buffer solution (1 M). During all the tests, the working electrode was rotated at 1600 rpm. A water jacket cell from Pine Research Instrumentation was used for all the tests to achieve controllable temperature.

## Supplementary information

Supplementary Information

Source data

Source data 2

## Data Availability

The data that support the findings of this study are available from the corresponding author upon request. The source data underlying Figs. 1a–d, 2a, 3a–c, 4a–c, and 5a, b and Supplementary Figs. 1–16 are provided as a Source Data file.
