# Supplementary material for: Anomalous hydrogen evolution behavior in high-pH environment induced by locally generated hydronium ions
Source: Nat Commun. 2019 Oct 25;10:4876. doi: 10.1038/s41467-019-12773-7 (PMC6814866; doi:10.1038/s41467-019-12773-7)
Supplement: Supplementary file 1 — Supplementary Information [file 41467_2019_12773_MOESM1_ESM.pdf]

Supplementary Information

Anomalous Hydrogen Evolution Behavior in High-pH Environment Induced by Locally Generated Hydronium Ions

Xuesi Wang[a‡], Chaochen Xu[a‡], Mietek Jaroniec[b], Yao Zheng[a]*and Shi-Zhang Qiao[a]*

[a] School of Chemical Engineering, The University of Adelaide, Adelaide SA 5005, Australia

[b] Department of Chemistry and Biochemistry, Kent State University, Kent, Ohio 44242, United States

E-mail: s.qiao@adelaide.edu.au; yao.zheng01@adelaide.edu.au

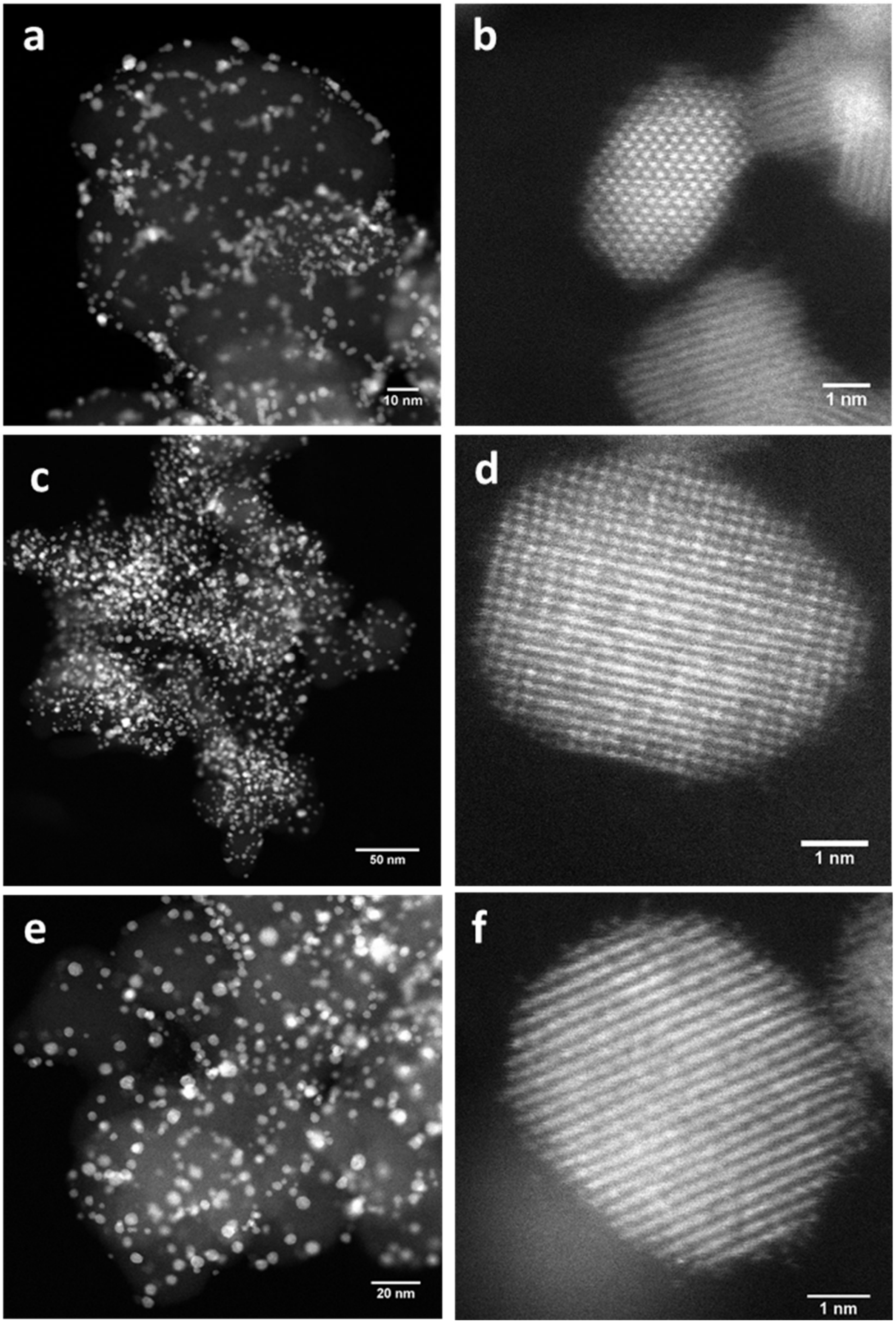

Supplementary Figure 1 The high-angle annular dark-field scanning transmission electron microscopy (HAADF-STEM) images of the samples. HAADF-STEM images of **a**, **b** Pt/C; **c**, **d** PtNi/C; **e**, **f** dealloyed PtCo/C nanosized electrocatalysts.

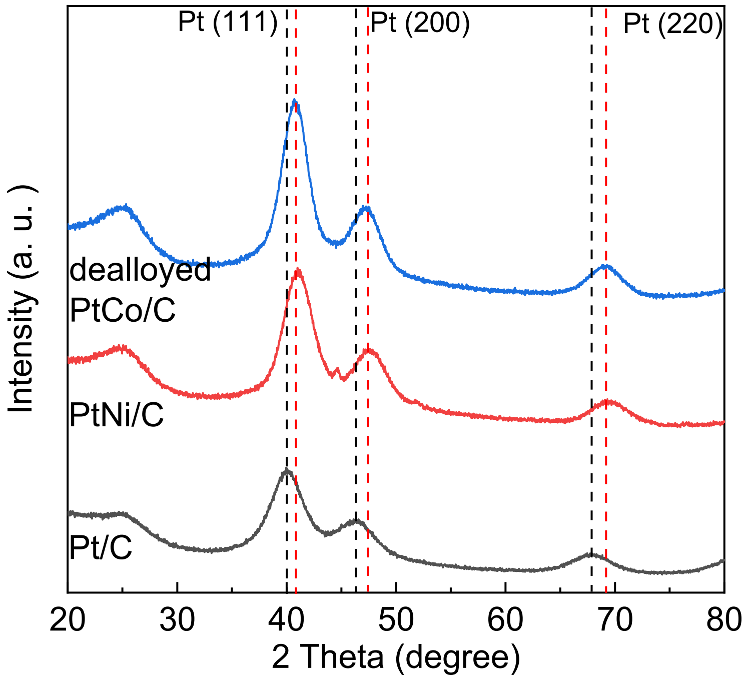

Supplementary Figure 2 XRD spectra of Pt/C, PtNi/C and dealloyed PtCo/C catalysts.

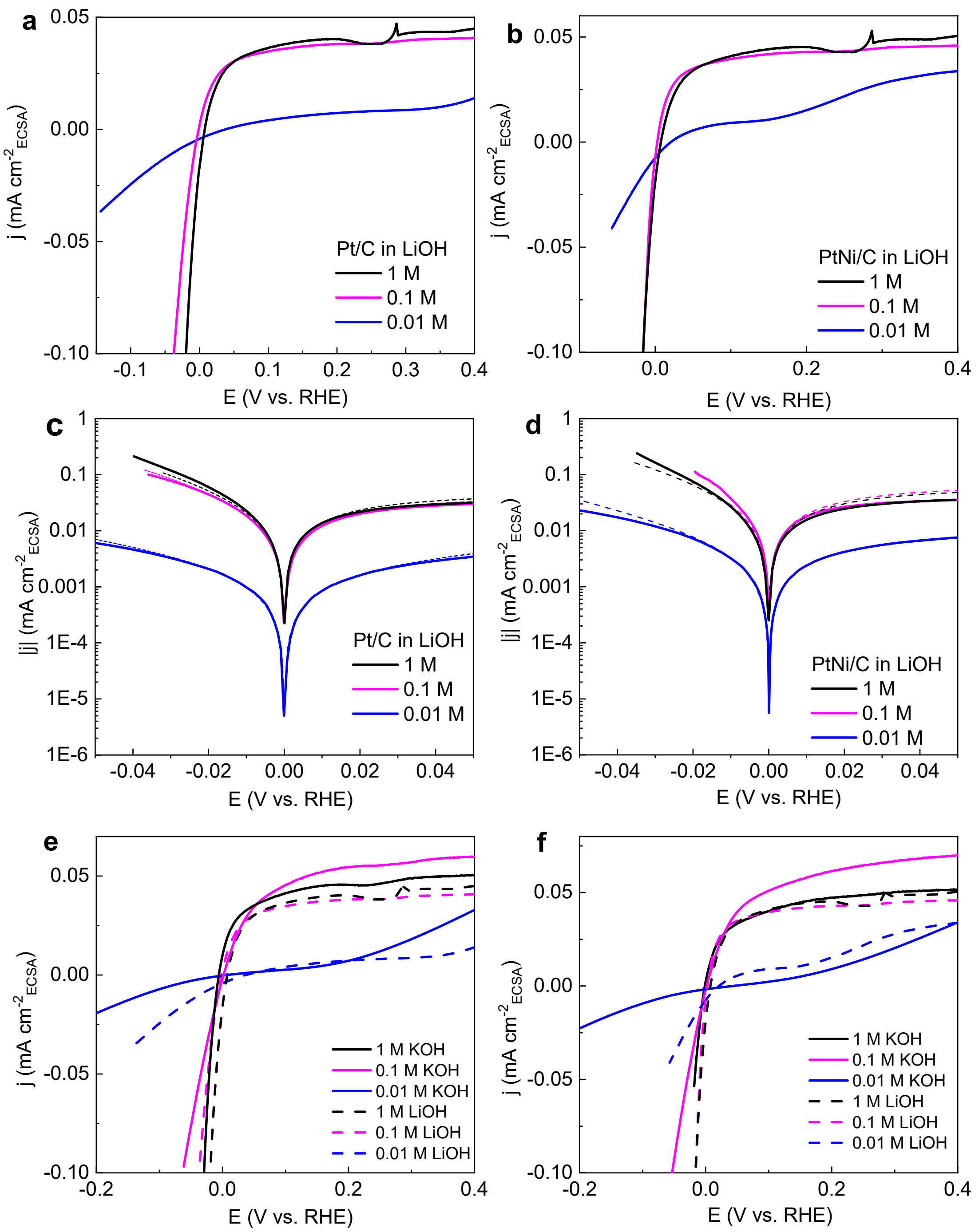

Supplementary Figure 3 The HER activity of the electrocatalysts. **a-b** The HER polarization curves measured for **a** Pt/C; **b** PtNi/C under different LiOH electrolytes. **c-d** The corresponding Butler-Volmer fitting results for **c** Pt/C; **d** PtNi/C under different conditions. **e-f** A comparison of the polarization curves measured for **e** Pt/C; **f** PtNi/C in two different electrolytes for a series of concentrations.

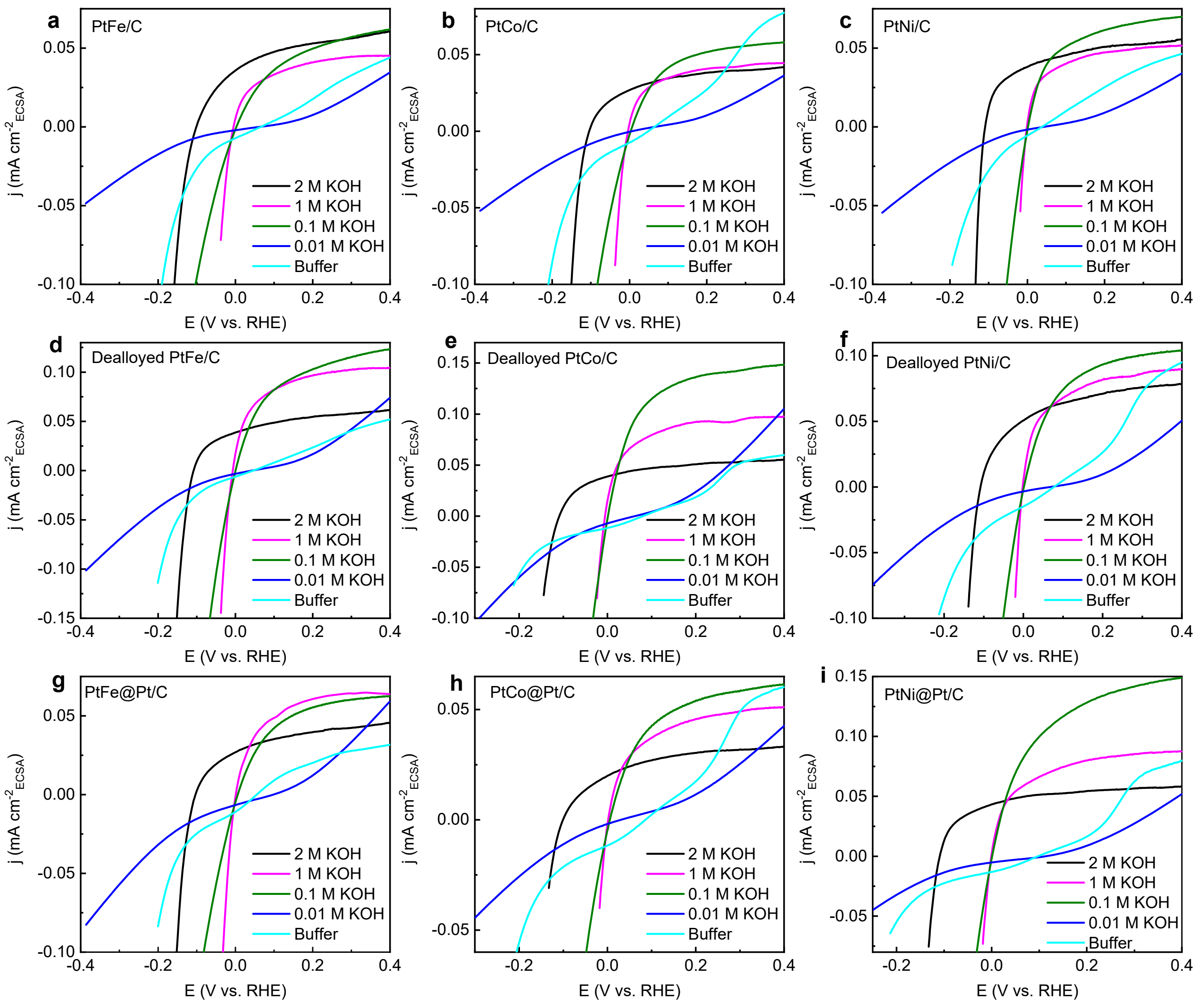

Supplementary Figure 4 The HER/HOR polarization curves obtained for a series of Pt-based nanosized electrocatalysts in different alkaline environments. The electrocatalysts are: **a** PtFe/C; **b** PtCo/C; **c** PtNi/C; **d** dealloyed PtFe/C; **e** dealloyed PtCo/C; **f** dealloyed PtNi/C; **g** PtFe@Pt/C; **h** PtCo@Pt/C; **i** PtNi@Pt/C.

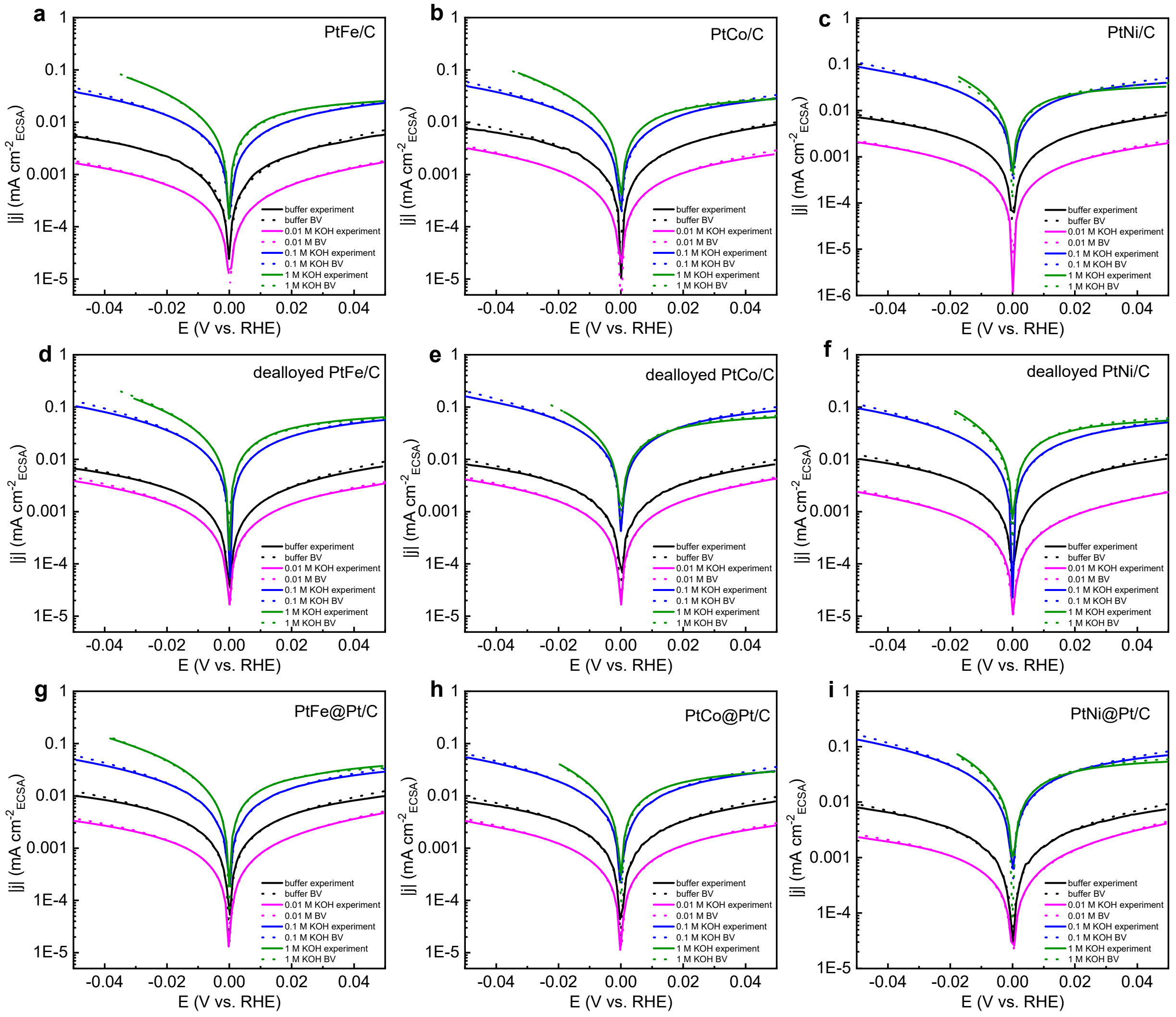

Supplementary Figure 5 The experimental Tafel plots and the corresponding Butler-Volmer fitting data (BV) for a series of Pt-based nanostructured electrocatalysts in different alkaline solutions. The electrocatalysts are: **a** PtFe/C; **b** PtCo/C; **c** PtNi/C; **d** dealloyed PtFe/C; **e** dealloyed PtCo/C; **f** dealloyed PtNi/C; **g** PtFe@Pt/C; **h** PtCo@Pt/C; **i** PtNi@Pt/C.

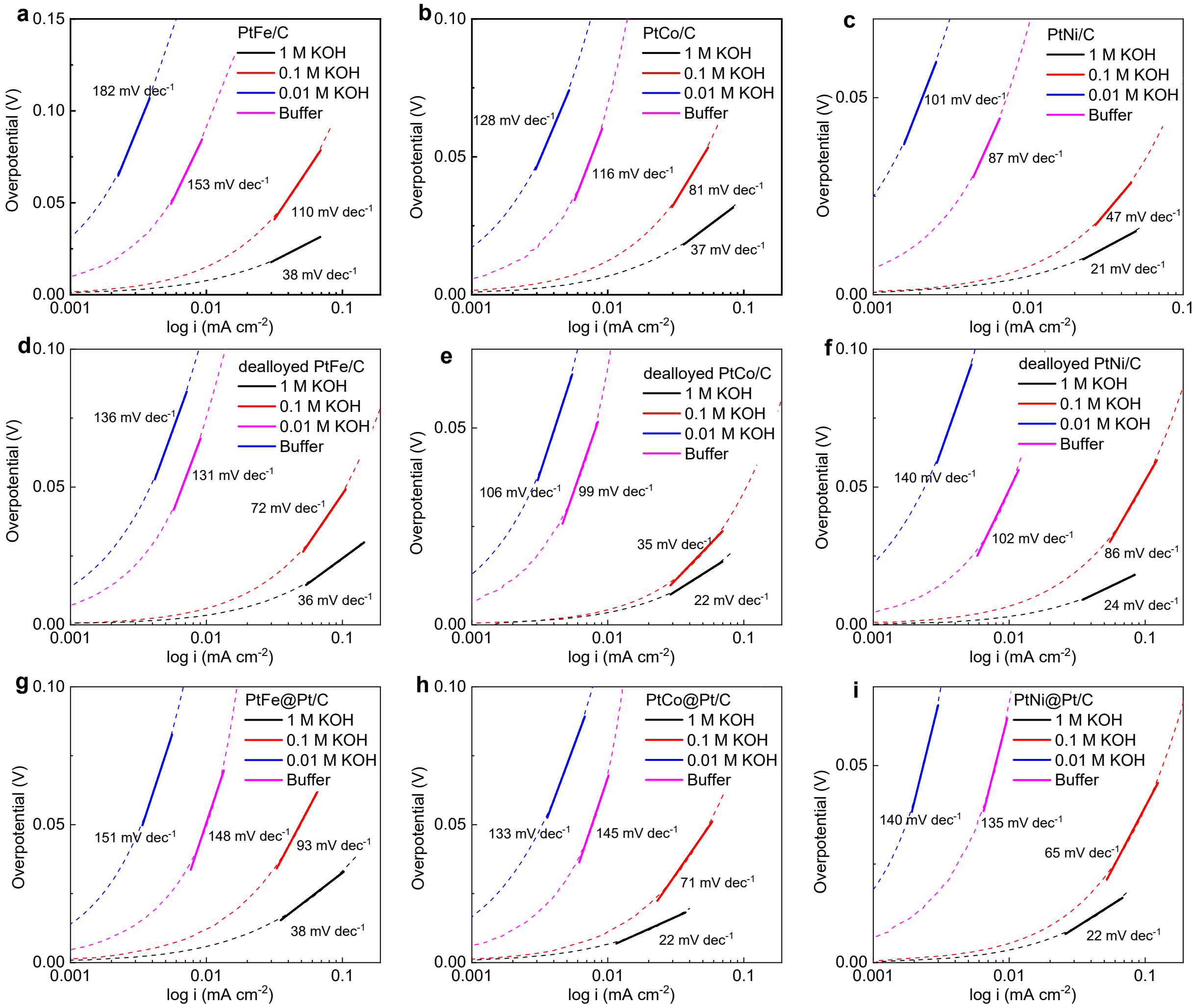

Supplementary Figure 6 Tafel slops for a series of Pt-based nanostructured electrocatalysts in different alkaline solutions. The electrocatalysts are: **a** PtFe/C; **b** PtCo/C; **c** PtNi/C; **d** dealloyed PtFe/C; **e** dealloyed PtCo/C; **f** dealloyed PtNi/C; **g** PtFe@Pt/C; **h** PtCo@Pt/C; **i** PtNi@Pt/C.

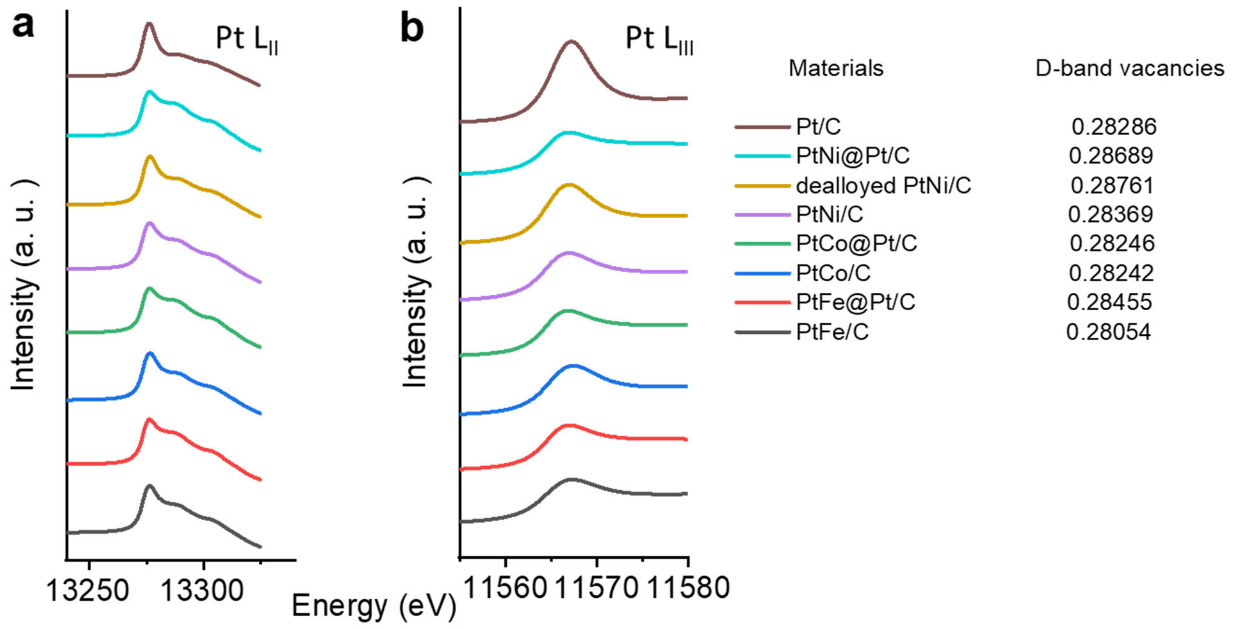

Supplementary Figure 7 The XANES spectra for a series of Pt-based nanosized electrocatalysts. The d-band vacancies were calculated based on the XANES data. **a** Pt L_{II} edge. **b** Pt L_{III} edge.

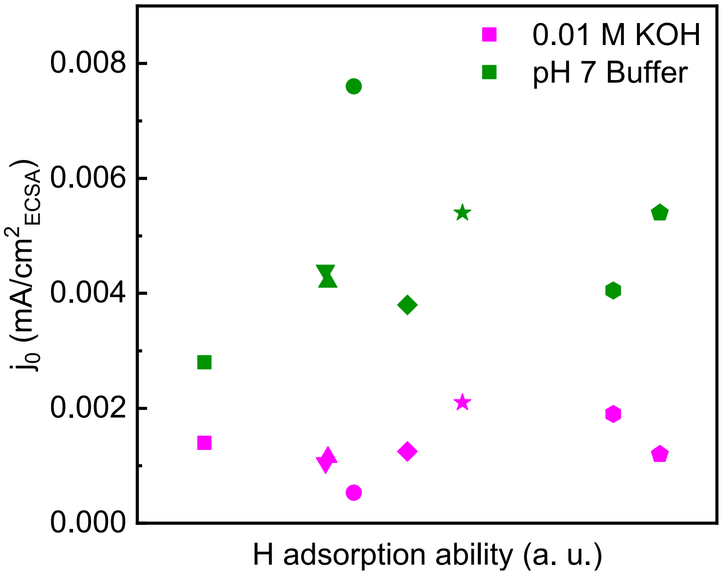

Supplementary Figure 8 The experimentally acquired relationship between the H adsorption ability and the activity (j_0) for a series of Pt based materials using different electrolytes. The H adsorption ability of the electrocatalysts is represented according to the d-band vacancies of each material. The symbols in the figure refers to **Fig. 2**.

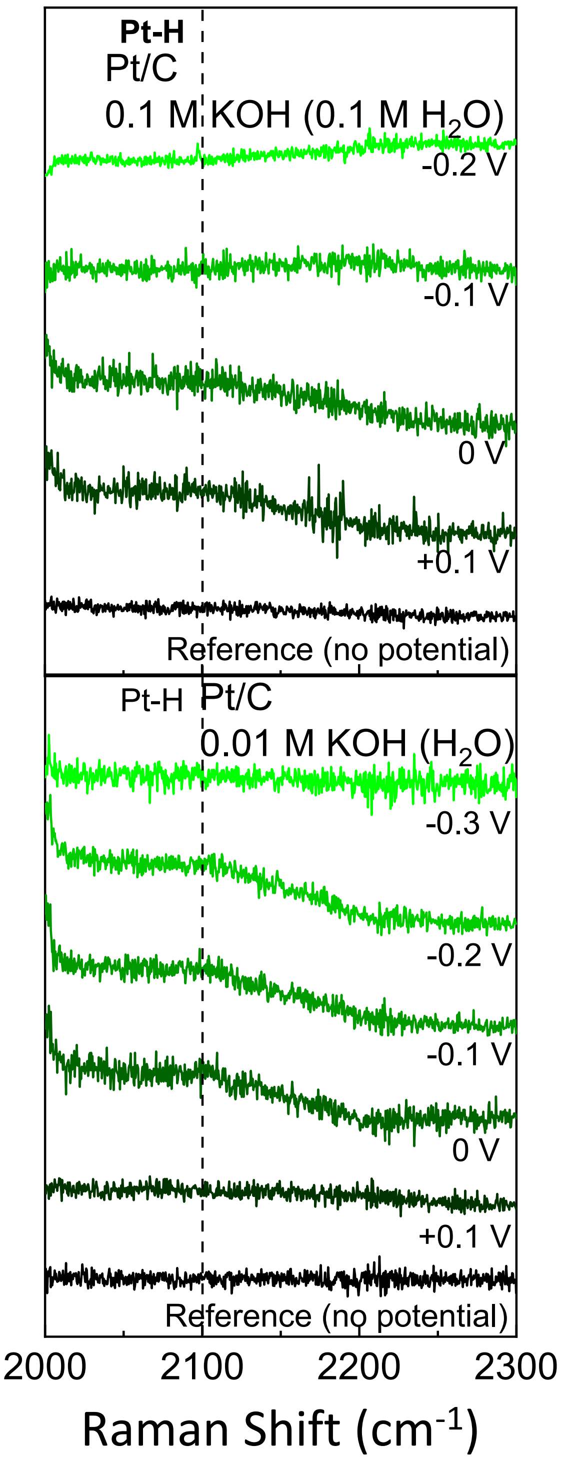

Supplementary Figure 9 Raman signal of Pt/C in different alkaline environments. Raman spectra of Pt/C in 0.1 M (above) and 0.01 M (below) KOH (H_2O) solutions at the Raman shift range of 2000-2300 cm^{-1}.

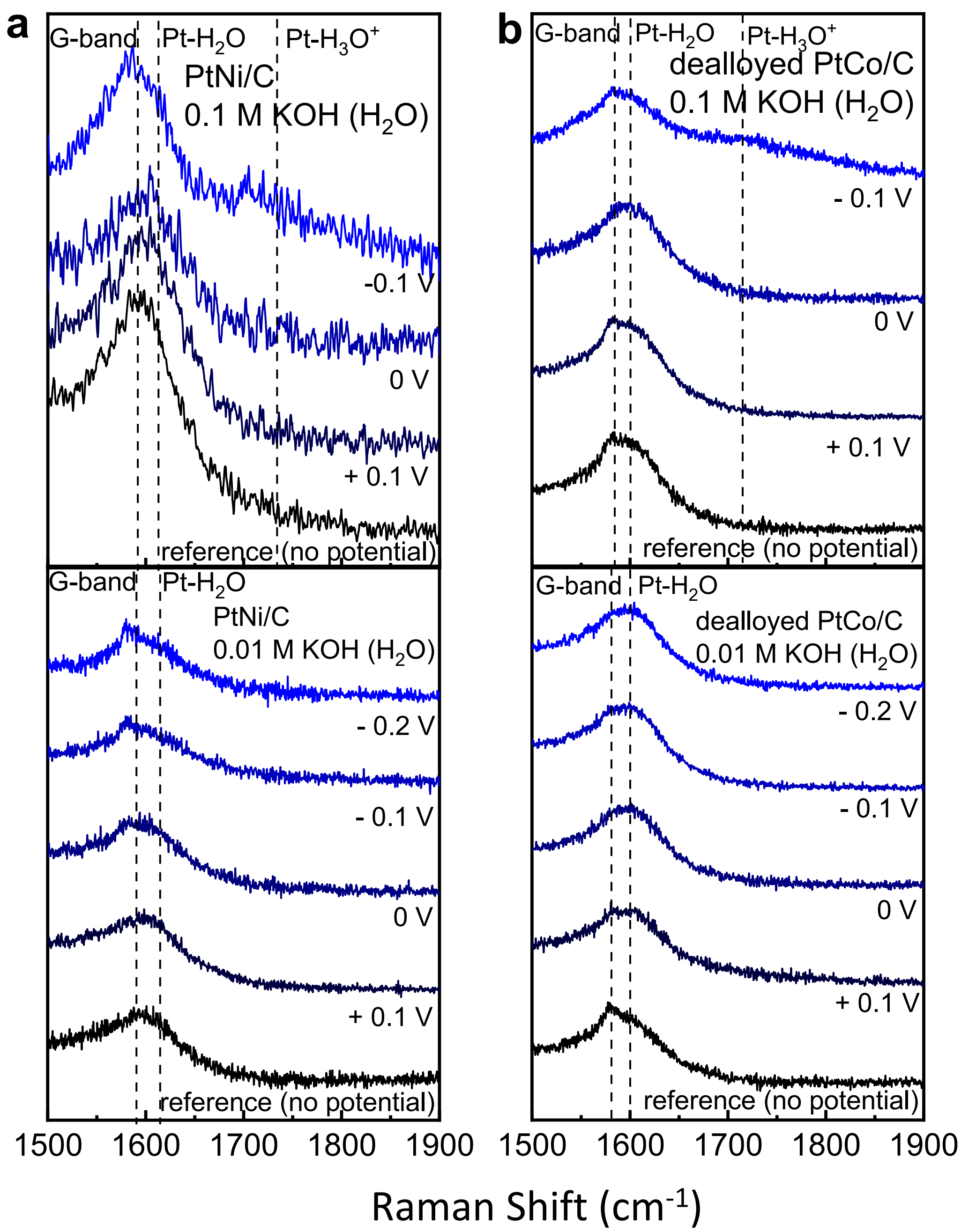

Supplementary Figure 10 Raman signal of different electrocatalysts. Raman spectra of: **a** PtNi/C in 0.1 M (above) and 0.01 M (below) KOH (H_2O), respectively. **b** dealloyed PtCo/C in 0.1 M (above) and 0.01 M (below) KOH, respectively.

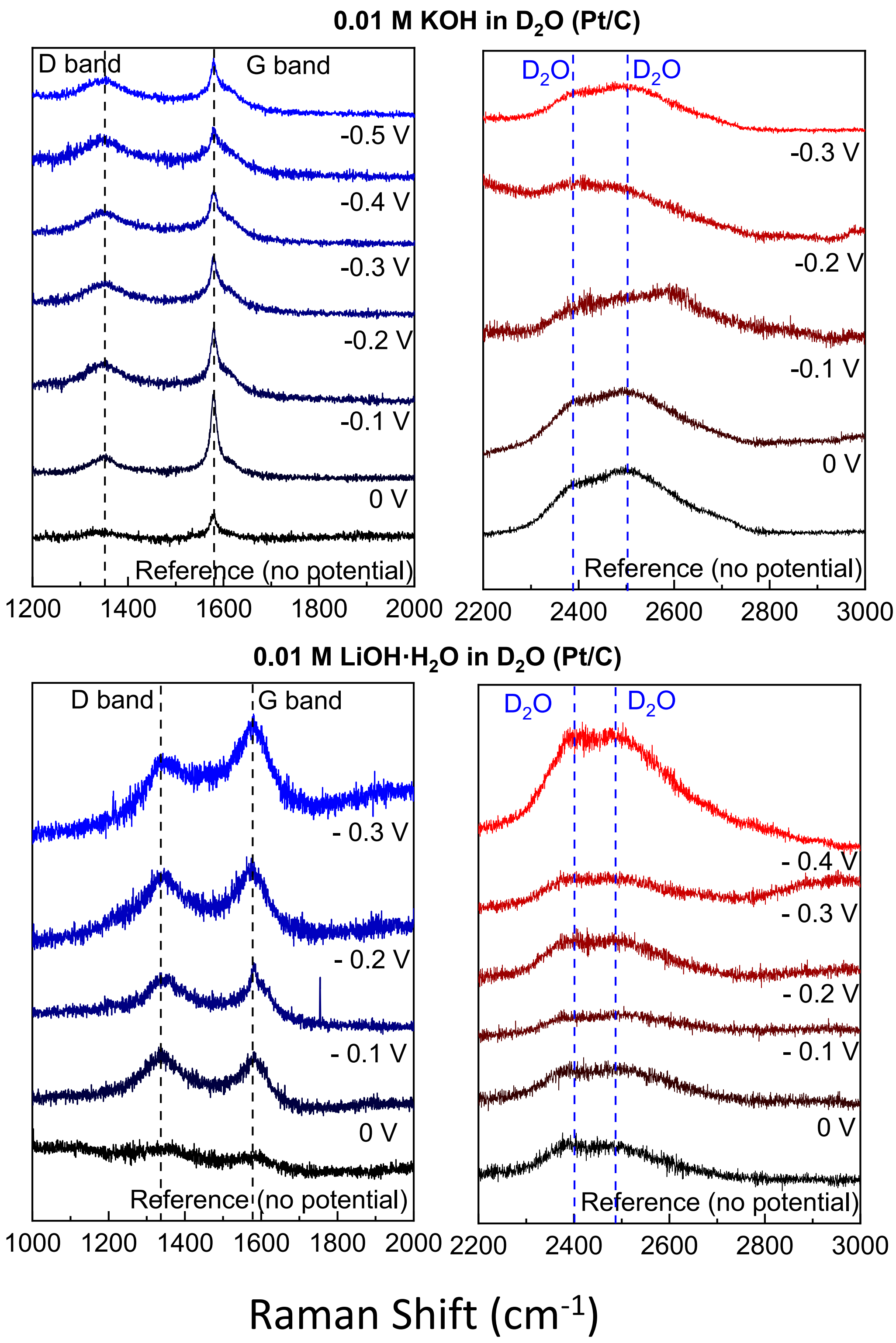

Supplementary Figure 11 Raman spectra of Pt/C in different environments. The electrolytes are 0.01 M KOH (top panels) and 0.01 M $LiOH\cdot H_2O$ (bottom panels) in D_2O solutions, respectively.

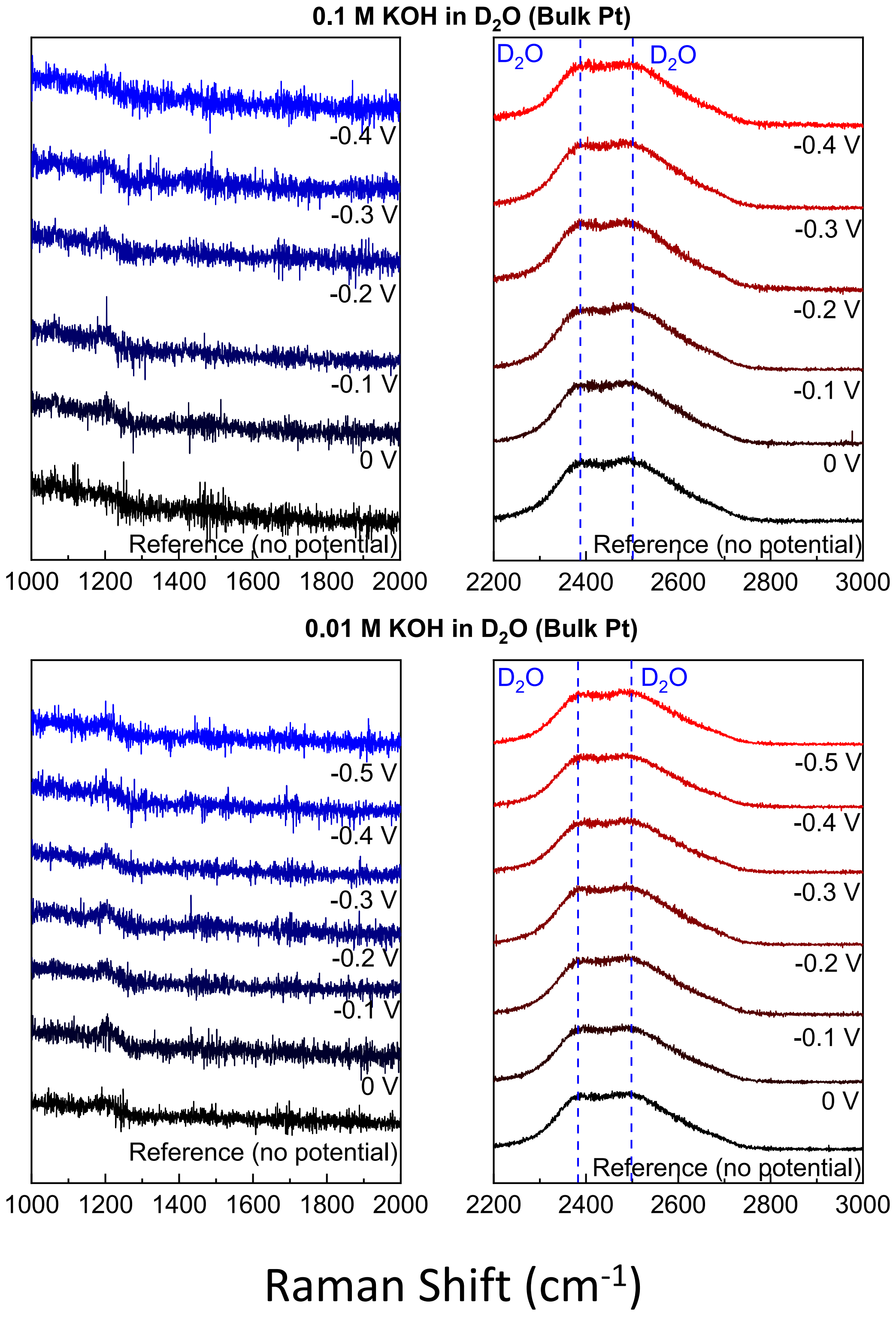

Supplementary Figure 12 Raman spectra of bulk Pt in different environments. The electrolytes are 0.1 M (top panels) and 0.01 M (bottom panels) KOH in D_2O solutions, respectively.

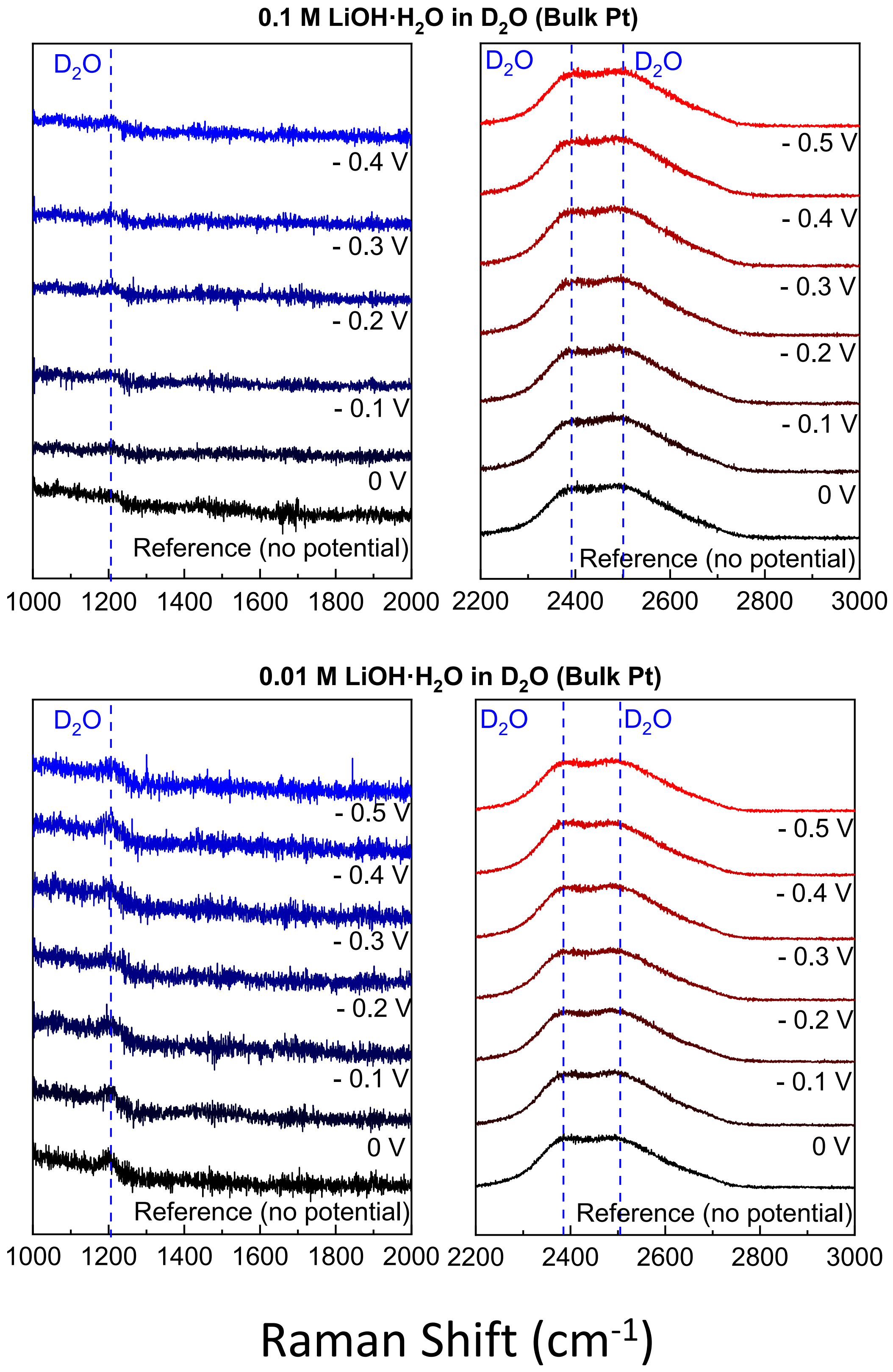

Supplementary Figure 13 Raman spectra of Pt/C in different environments. The electrolytes are 0.1 M (top panels) and 0.01 M (bottom panels) $LiOH \cdot H_2O$ in D_2O solutions, respectively.

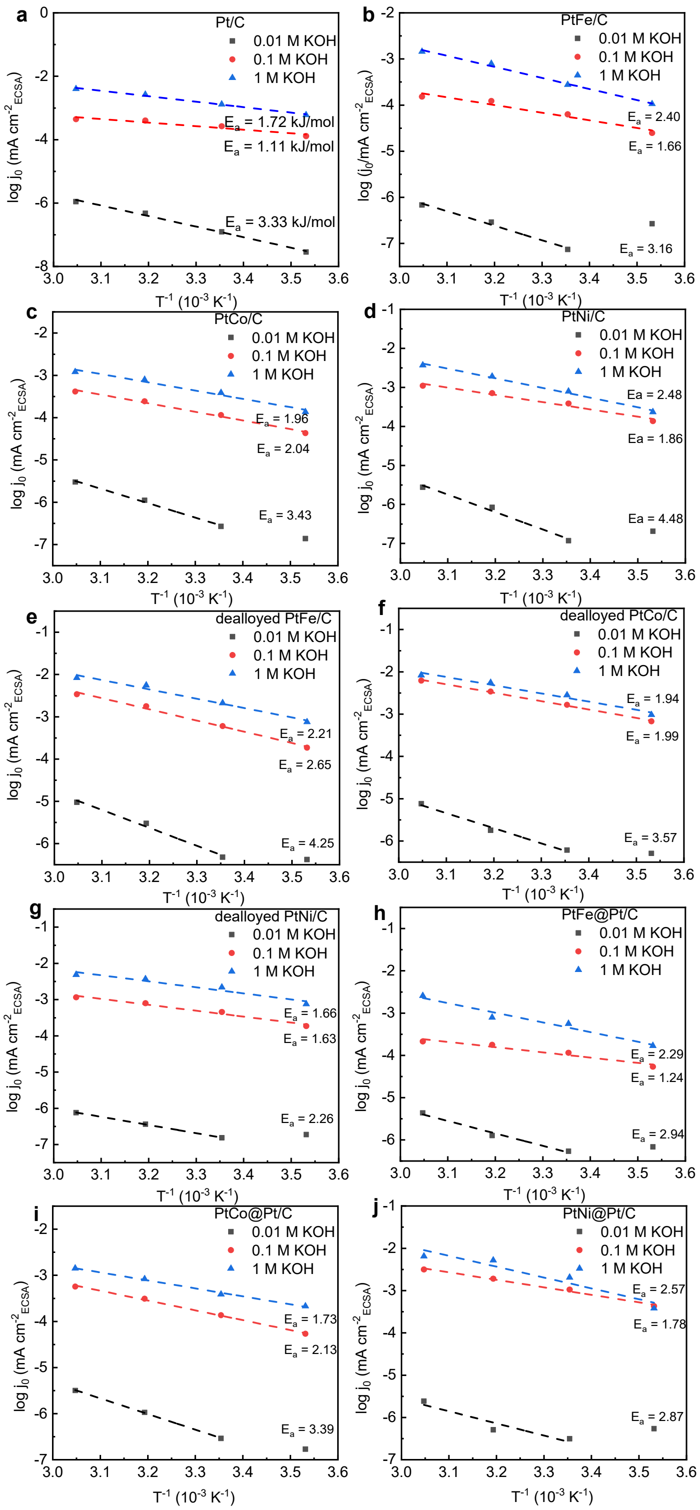

Supplementary Figure 14 The activation energy calculated using the relationship between j_0 and temperature in different alkaline environments for a series of Pt-based electrocatalysts. The electrocatalysts are: **a** Pt/C; **b** PtFe/C; **c** PtCo/C; **d** PtNi/C; **e** dealloyed PtFe/C; **f** dealloyed PtCo/C; **g** dealloyed PtNi/C; **h** PtFe@Pt/C; **i** PtCo@Pt/C; **j** PtNi@Pt/C.

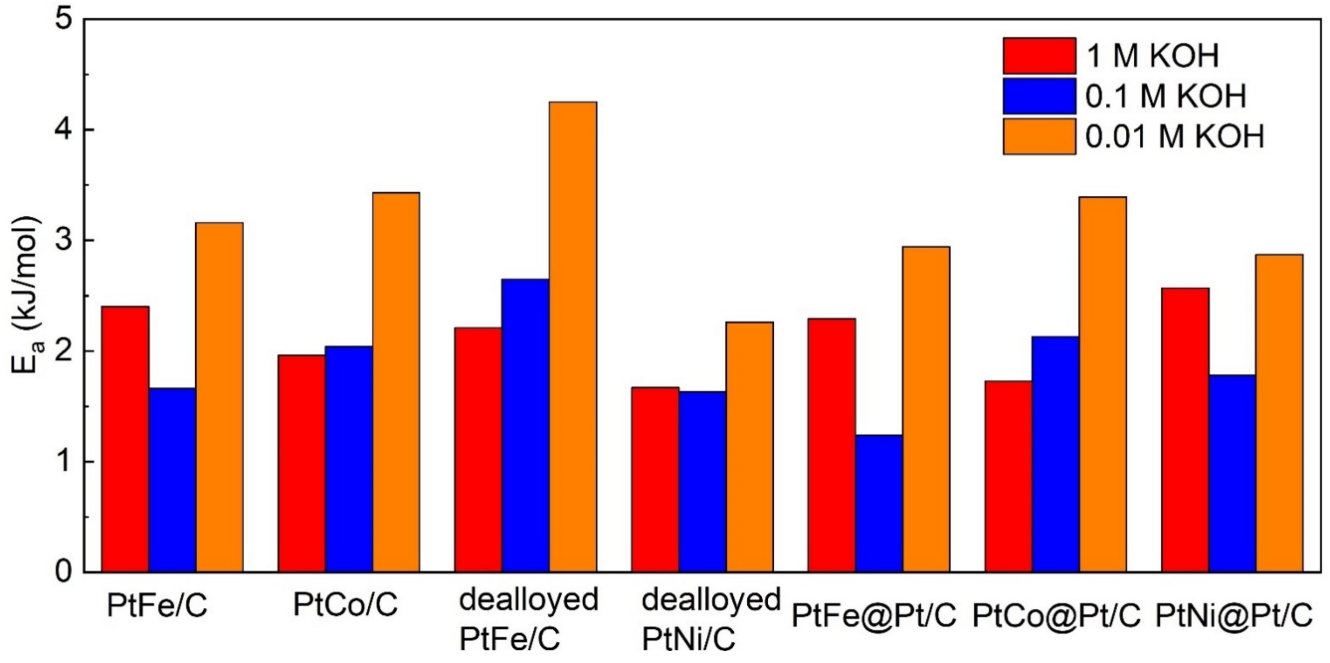

Supplementary Figure 15 A comparison of E_a for a series of different Pt-based nanosized electrocatalysts in different alkaline environments.

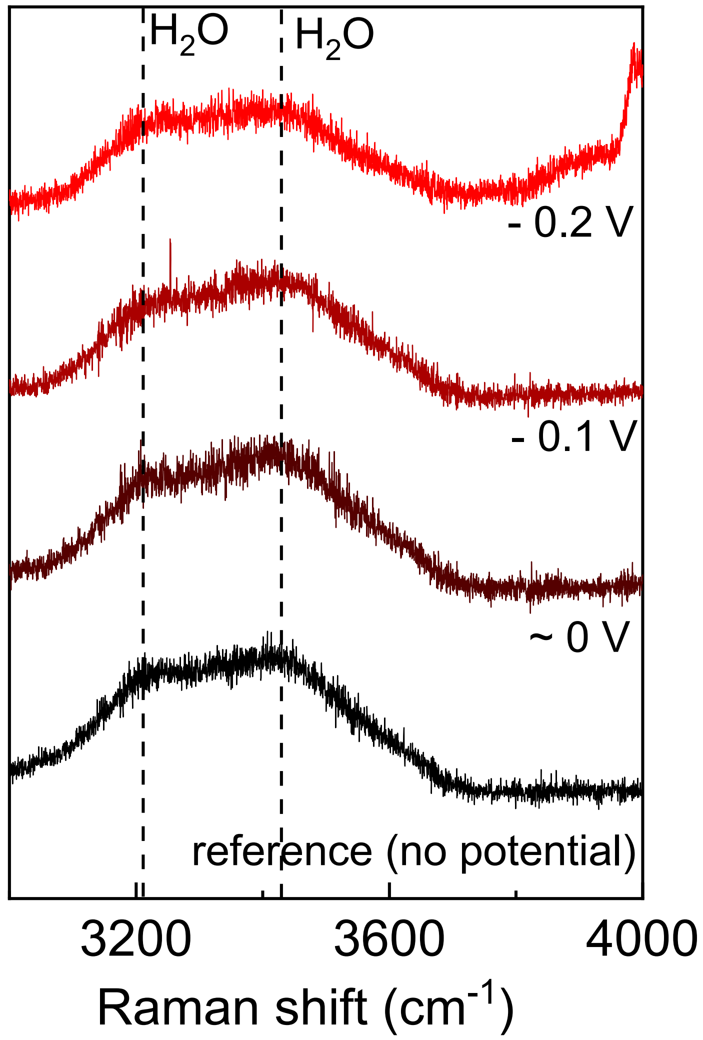

Supplementary Figure 16 Raman spectra of dealloyed PtCo/C in 0.1 M KOH aqueous solution.
